# From debulking to delivery: sequential use of rotational atherectomy and Guidezilla™ for complex saphenous vein grafts intervention

**DOI:** 10.1186/s12872-018-0860-y

**Published:** 2018-06-19

**Authors:** Mariano Pellicano, Vincent Floré, Emanuele Barbato, Bernard De Bruyne

**Affiliations:** 1 Cardiovascular Center Aalst, OLV Hospital, Moorselbaan 164, B 9300 Aalst, Belgium; 2 Department of Advanced Biomedical Sciences, Federico II University of Naples, Via Pansini, 5, 80131 Naples, Italy

**Keywords:** Saphenous vein graft, Rotational atherectomy, Percutaneous coronary intervention, Non-ST segment elevation myocardial infarction, Local stent delivery technique

## Abstract

**Background:**

Percutaneous coronary interventions (PCI) of old calcified saphenous vein grafts (SVGs) is challenging and is associated with a considerably high risk of adverse ischemic events in the short- and long-term as compared to native coronary arteries. We report a case in which a non-dilatable, calcified SVG lesion is successfully treated with rotational atherectomy followed by PCI and stenting with local stent delivery (LSD) technique using the Guidezilla™ guide extension catheter (5-in-6 Fr) in the “child-in-mother” fashion.

**Case presentation:**

A 70 years-old man with a dilated ischemic cardiomyopathy, triple coronary artery bypass grafting (CABG) in 1990 and chronic renal failure (baseline GFR: 45 ml/min/1.73 m^2^) underwent a coronary angiography for a Non-ST segment elevation myocardial infarction (NSTEMI). Native coronary circulation was completely occluded at the proximal segments. Grafts angiography showed a tandem calcified lesions of SVG on distal right coronary artery (RCA) and an ostial stenosis of the SVG on first obtuse marginal branch (OM1). Left internal mammary artery on the mid left anterior descending artery was patent. Ad Hoc PCI of SVG on RCA was attempted. The proximal calcified stenosis has been crossed with a 1.5 x 12 mm balloon only with the support of Guidezilla™, however the non-compliant (NC) balloon 2.5 x 15 mm was unable to break the hard and calcified plaque. After several attempts, the procedure was interrupted with a suboptimal result. An elective transradial PCI of SVG on RCA with rotational atherectomy was performed. Two runs with 1.25 mm burr and 2 runs with 1.5 mm burr were carried out. Then, the use of distal anchoring balloon warranted support and tracking, made as centring rail for the advance of the tip of the “mother-and-child” catheter into the SVG. During slow deflation of the balloon, the Guidezilla™ was advanced distal to the stenoses to be stented, thus allowing the placement of two long drug eluting stents according to a LSD technique.

**Conclusions:**

Rotational atherectomy is a feasible option for non-dilatable stenoses in old SVGs when there is no evidence of thrombus or vessel dissection and the subsequent use of “mother-and-child” catheter has a key role, especially in case of radial approach, for long stents delivery.

## Background

Stent delivery with severe coronary calcifications and tortuosities is still a common cause of percutaneous coronary interventions (PCI) failure in both native coronary arteries and bypass grafts [1]. PCI of old calcified saphenous vein grafts (SVGs) is challenging and is associated with a considerably high risk of adverse ischemic events in the short- and long-term as compared to native coronary arteries [2–4]. Up to date the use of rotational atherectomy device in calcified SVGs still remain an “off-label” indication [5–7]. We report a case in which a non-dilatable, calcified SVG lesion is successfully treated with rotational atherectomy followed by PCI e stenting with local stent delivery (LSD) technique using the Guidezilla™ guide extension catheter (5-in-6 Fr) in the “child-in-mother” fashion.

## Case presentation

**Fig. 1:**
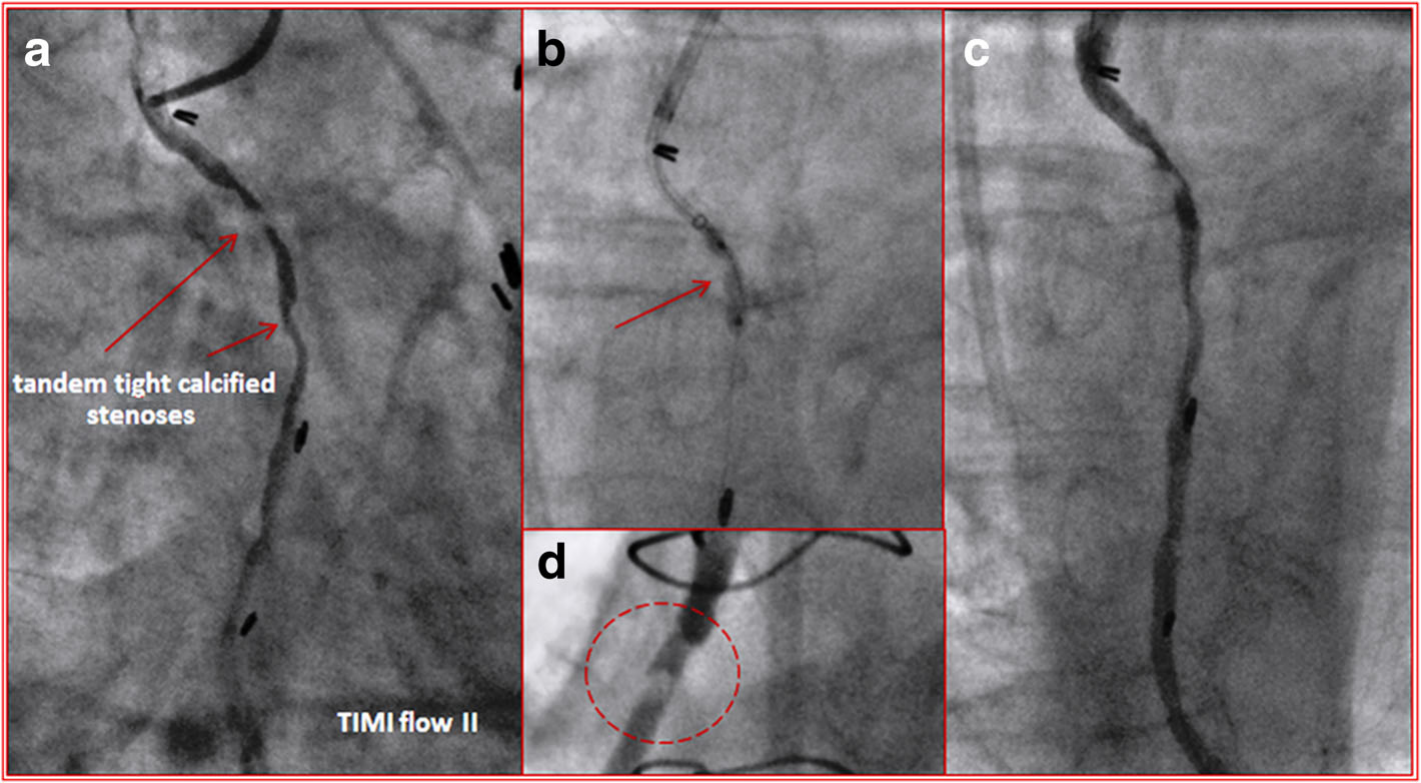
**a** fist injection of SVG-RCA; tandem calcified lesions (arrows); **b** balloon 2.5 × 15 mm unable to break the proximal calcified plaque (arrow); **c** unsuccessful final result of the first procedure; **d** proximal calcified stenosis after several predilations (dashed circle)

**Fig. 2:**
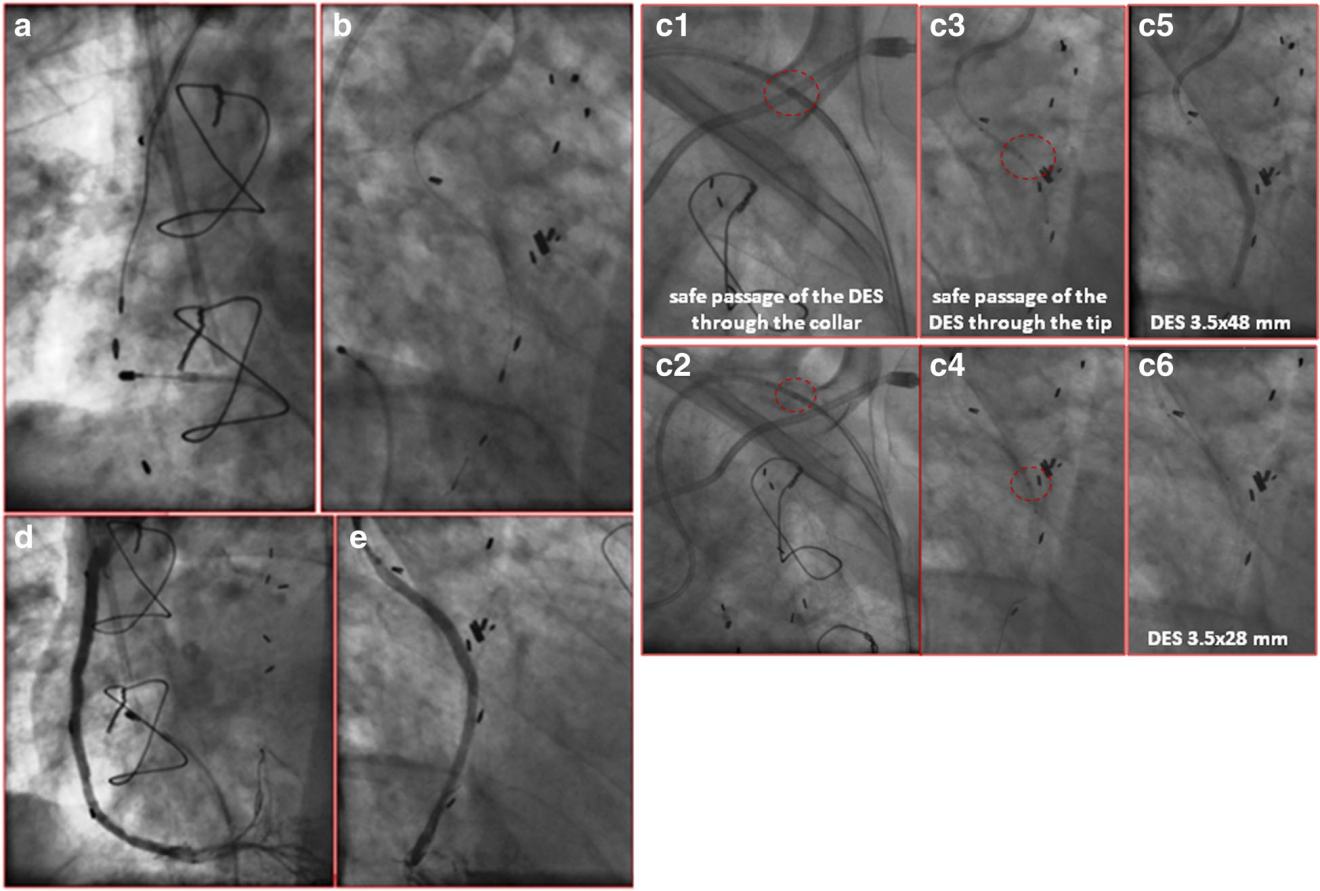
**a** runs with 1.25 mm burr and **b** 1.5 mm burr; **c** step by step local stent delivery technique using anchoring balloon and Guidezilla™ for the placement of 2 DES (3.5 × 48 mm and 3.5 × 28 mm); **d** and **e** final result

A 70 years-old man with a dilated ischemic cardiomyopathy, triple coronary artery bypass grafting (CABG) in 1990 and chronic renal failure (baseline GFR: 45 ml/min/1.73 m^2^) underwent a coronary angiography for a Non-ST segment elevation myocardial infarction (NSTEMI). Baseline values of Hs-Troponin T and CK-MB were 497 ng/l and 211 U/l respectively. Native coronary circulation was completely occluded at the proximal segments. Grafts angiography showed a tandem calcified lesions of SVG on distal right coronary artery (RCA) (Fig. 1a) and an ostial stenosis of the SVG on first obtuse marginal branch (OM1). Left internal mammary artery on the mid left anterior descending artery was patent. Ad Hoc PCI of SVG on RCA was attempted. The proximal calcified stenosis has been crossed with a 1.5 x 12 mm balloon only with the support of Guidezilla™ guide extension catheter (5-in-6 Fr), however the non compliant (NC) balloon 2.5 x 15mm was unable to break the hard and calcified plaque (Fig. 1b). After several attempts, the procedure was interrupted with a suboptimal result (Fig. 1c and d). An elective PCI of SVG on RCA with rotational atherectomy was performed (left radial approach, 6 French). Two runs with 1.25 mm burr (Fig. 2a) and 2 runs with 1.5 mm burr (Fig. 2b) were carried out. Then, the use of distal anchoring balloon warranted support and tracking, made as centring rail for the advance of the tip of the “mother-and-child” catheter into the SVG. During slow deflation of the balloon, the Guidezilla™ catheter (5-in-6 Fr) was advanced distal to the proximal stenosis to be stented, thus allowing a first drug eluting stent (DES) 3.5 × 48 mm to be placed on the mid-distal segment of the graft according to a LSD technique. The same technique was applied to deploy a second DES proximally to the first one (3.5 × 28 mm on proximal segment). During both rotational atherectomy and PCI, as well as at the final angiographic control, we did not observe any sign of embolization and the procedure was successfully completed with a final TIMI III flow without complications. A slight elevations of the cardiac biomarkers the day after the procedure (Hs-Troponin T 564 ng/l and CK-MB 308 U/l) was followed by a gradual reduction of the latters in the next 4 days and the patient was discharged in stable conditions.

## Discussion and conclusions

SVG calcification occurs more commonly in older grafts of patients with advanced age and kidney disease, insulin-dependent diabetes mellitus and in smokers. It is a process that occurs mainly within the wall and not within the plaque, that leads to a significant structural changes with arterialization and (passive or active) degeneration of SVG [8]. Currently available evidence of the use of rotational atherectomy in calcified SVG are limited to small case series and few case reports [9]. Thomas WJ et al. reported the safe and effective use of rotational atherectomy to facilitate PCI and stenting in 14 patients with SVG stenoses. However this experience was primarily focused on treatment of anastomotic sites (aorto-ostial and distal), and restenotic or fibrotic undilatable lesions rather than calcified non-dilatable stenoses in the body of old SVGs [10]. Rotational atherectomy is not frequently used if the stenoses are located in the body of the SVGs because of the concern of soft atherosclerotic or thrombotic material that is friable and compliant, with high risk of embolization and no-reflow phenomenon. However the use of rotational atherectomy in the body of calcified SVGs, performed by experienced operators, seems to be an attractive option because of the potential advantage of rotablation over balloon angioplasty and cutting balloon in terms of debulking strategy that would allow for stent placement. In SVG intervention, distal protection with a balloon-occlusion/aspiration systems or a filter-based device have proven clinical benefits and should be used when technically feasible. However anatomic, technical, and operator-related issues may reduce the effectiveness of the distal protection devices. In our case, because of the complexity of the tandem calcified lesions of the SVG a distal filter could not be advanced in the first procedure and, in the second procedure, since the rotational atherectomy was considered as a primary choice, the placement of a distal filter was incompatible with our strategy.

While some limited experiences of rotational atherectomy in SVGs are reported in the literature, no data regarding usefulness, safety and feasibility of the simultaneous or sequential (as in our case) use of mother-and-child catheter and rotablator in bypass grafts are available, and this is the peculiarity of our case.

The Guidezilla™ 5-in-6 Fr is a mother-and-child catheter with an inner lumen minimally larger than the corresponding GuideLiner™ (0.057″ instead of 0.056″), with a hypotube rather than a steel ribbon to improve pushability, a proprietary coating rather than silicone and with a polymer coated metal collar to facilitate device insertion at the transition point [11]. In complex SVGs interventions, the use of Guidezilla™ should be considered in order to: [1] improve the coaxial engagement of SVGs ostia, which have a shallow take-off from the ascending aorta, [2] maximize guiding catheter (GC) backup with the “swan neck” technique, in which the extension engages the graft while the GC stands outside and is pushed against the aortic wall and, finally, [3] ensure the distal stent delivery [12]. In a prospective analysis of Chan PH et al. regarding the extended use of the GuideLiner™ in complex coronary interventions, 8 out of 55 patients included underwent PCI of SVG lesions and 5 of these had chronic occlusion (CTO) of the SVG. In all the cases the use of mother-and-child catheter guaranteed the procedural success [13]. Similarly De Man FHAF et al. prospectively evaluated in a series of 64 patients the usefulness and safety of the GuideLiner™ (Twente GuideLiner registry). All the cases for which the SVG was the target vessel (14% - 10/70 lesions) were successfully performed without major and minor complications [14].

Our case shows the importance of sequential use of Rotablator and Guidezilla™, the first for debulking and plaque modification purpose and the second for delivery purpose. The basic principle behind plaque modification of heavily calcified and undilatable lesions with rotational atherectomy is to debulk the offending plaque rather than fracturing it or displacing it [5]. This is crucial in order to facilitate the subsequent delivery of long stents, especially in case of a transradial PCI of vein grafts [12]. Indeed in old calcified SVGs stenoses, despite the use of multiple shapes of GCs, adequate support for guidewire advancement and the passage of balloon catheters and stents could not be readily achieved, as occurred in our first procedure.

In this report we also propose the “LSD” technique, in which we systematically proceed through the following steps: 1) insertion of the guidewire into the coronary artery; 2) advancement of the Guidezilla™ as close to the lesion as possible; 3) inflation of the balloon in the stenosis; 4) while slowly deflating the balloon, the Guidezilla™ catheter is placed across and distal to the stenosis (‘telescope maneuver’); 4) placement of the stent distally to the stenosis; 5) pull back of the Guidezilla™; 6) positioning and deployment of the stent in the segment. In this case, after rotational atherectomy we performed several predilations with NC balloons 3 × 15 mm and 3 × 20 mm, keeping the RotaWire is 0.009′′ (Boston Scientific, Marlborough, MA, USA) distally into the postero-lateral branch. The technique allows to avoid aggressive manipulations of the GC as well as the use of buddy wires and ensures the placement of one long stent instead of multiple stents implantation in overlapping. In our case, because of the severe and diffuse atherosclerotic disease and the substantial length of rotational atherectomy in the SVG, we implanted the two longest DES available on the shelf (3.5 × 48 mm in mid-distal segment and 3.5 × 28 mm in proximal segment). Avoiding the need for multiple stents (and hence the risk of failure to overlap) by maximising the length of the individual stents used may assist in the avoidance of geographic miss. Moreover multiple stents implantation is associated with an higher rate of death, acute myocardial infarction (MI) and major adverse cardiac events at 30 days and 6 months follow-up as compare to single stent implantation [15, 16]. Especially in diabetic patients with complex coronary or bypass grafts lesions, PCI with multiple stents should be undertaken with great caution. Although stent length is an important predictor of adverse events after PCI, second generation everolimus-eluting stent (EES) for the treatment of long lesions seems to be safe and efficient, with a low rate of target lesion revascularization (TLR) together with a low rate of death and MI, in both native coronary arteries [17–19] and SVGs [20].

In conclusion, rotational atherectomy is a feasible option for non-dilatable stenoses in old SVGs when there is no evidence of thrombus or vessel dissection and the subsequent use of “mother-and-child” catheter has a key role, especially in case of radial approach, for long stents delivery.

## Abbreviations

CABG: Coronary artery bypass grafting
CTO: Chronic total occlusion
DES: Drug eluting stent
EES: Everolimus-eluting stent
GC: Guiding catheter
LSD: Local stent delivery
MI: Myocardial infarction
NC: Non-compliant
NSTEMI: Non-ST segment elevation myocardial infarction
OM: Obtuse marginal branch
PCI: Percutaneous coronary interventions
RCA: Right coronary artery
SVG: Saphenous vein graft
TLR: Target lesion revascularization
